# Intestinal obstruction secondary to torsion of an appendix epiploica: a case report

**DOI:** 10.4076/1757-1626-2-6475

**Published:** 2009-07-30

**Authors:** Kasim A Behranwala, Tushar Agarwal, Charlotte Treacy, Avril Chang

**Affiliations:** General Surgery Department, Central Middlesex HospitalActon Lane, Park Royal, NW10 7NS and Imperial College, LondonUK

## Abstract

**Introduction:**

Appendices epiploicae are affected by spontaneous torsion, calcification, primary or secondary inflammation, enlargement by lipomas or metastases and incarceration in hernias.

**Case presentation:**

A 20-year-old Asian man was admitted with non-specific abdominal pain, which later evolved to intestinal obstruction. Operative findings showed the small bowel obstruction was due to an omental band adhered to a nodule. Histopathology of the nodule revealed an infarcted appendix epiploica.

**Conclusion:**

Heightened suspicion and increased awareness of this entity would have led to an earlier diagnosis. Acute torsion of an appendage usually manifests as localised abdominal pain in one of the lower quadrants. Untreated, peritonitis or intestinal obstruction may result. Use of diagnostic laparoscopy in non-resolving abdominal pain would help to resolve the issue at an earlier stage and prevent additional morbidity.

## Introduction

Appendices epiploicae are adipose structures protruding from the serosal surface of the colon. Approximately 50-100 is present normally and is arranged in two separate longitudinal rows extending from the caecum to the rectosigmoid junction. They are thought to have a protective function, similar to that of the greater omentum, containing any intra-abdominal infection. They appear as lobulated masses of pericolic fat, usually 2-5 cm long and 1-2 cm thick on abdominal radiography, CT scan or US, if intraperitoneal contrast material, ascites or blood surrounds the colonic wall. The use of intraperitoneal gas as a negative contrast agent is apparently inadequate for radiologic demonstration of the appendage. The radiologic findings (contrast radiography, CT and US) provide useful criteria for preoperative recognition of torsion, infarction and primary inflammation of appendices epiploicae [1].

Appendices epiploicae are affected by spontaneous torsion followed by ischaemic or haemorrhagic infarct, calcification due to aseptic fat necrosis, primary or secondary inflammation, enlargement by lipomas [2] or metastases and incarceration in hernias. Non-specific clinical signs and symptoms (eg. torsion is mistaken for appendicitis or diverticulitis) often manifest disorders of appendices epiploicae. These entities should be included in the differential diagnosis of any unexplained abdominal pain or pericolic lesions in adults [1].

Each epiploic appendage is supplied by one or two small endarteries branching from the vasa recta longa of the colon and drained by a rather tortuous vein passing through its narrow pedicle. Such a limited blood supply, together with their pedunculated shape and excessive mobility, makes appendices epiploicae prone to torsion and ichaemic or haemorrhagic infarct [3].

## Case presentation

A 20-year-old Asian man was admitted with a three day history of central abdominal pain and decreased appetite but no history of vomiting or constipation. Examination of his abdomen revealed mild tenderness to the right of the umbilicus and bowel sounds were normal. All blood results including inflammatory markers were normal and an abdominal X-ray did not show anything unusual. He was started on conservative management.

**Figure 1.**
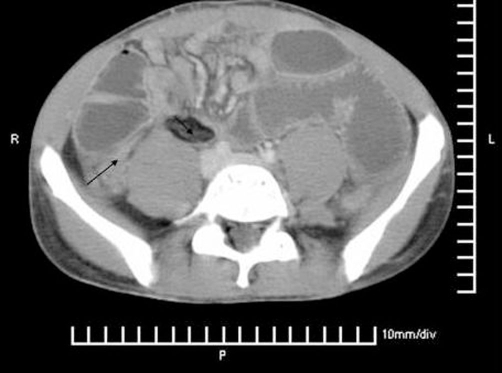
Preoperative CT scan showing acute intestinal obstruction with small bowel dilatation (arrow points towards the mass caused by the inflammation of the appendices epiploic).

The next day, the patient’s abdominal pain increased. He was tender in both the iliac fossae on clinical examination with no rebound tenderness or guarding. He however settled down by evening and was well the next day. The following day (4th day since admission), the pain recurred and he started vomiting. He was passing flatus but no faeces. On examination, he had a slightly distended abdomen and was tender on the right side of his abdomen. There was no guarding or rigidity. Blood tests showed rising inflammatory markers (neutrophil leucocytosis) (White cell count - 12.2 and C - reactive protein 22). An abdominal X-ray showed dilated small bowel loops. He proceeded to have a CT scan of his abdomen which showed evidence of small bowel obstruction with a cut off at the mid ileum (Figure 1).

In view of his clinical picture, a decision was taken to perform a diagnostic laparoscopy. This however had to be converted to a laparotomy due to significant dilatation of small bowel loops. Operative findings included dilated small bowel loops till the mid-ileum where an ischaemic stricture with perforation of the small bowel had resulted as a consequence of an omental band. This omental band had encircled the small bowel and was adherent to a nodule arising from one of the appendices epiploicae. The appendix was slightly congested. The band was divided and a wedge resection of the small bowel was carried out. The nodule to which the omental band had stuck to was excised and sent for histology. The appendix was removed.

The patient developed a small pelvic collection post -operatively which was managed conservatively with antibiotics. He was discharged from hospital 12 days after surgery and did not have any further complications.

The histology of the excised nodule showed an infarcted appendix epiploicae. Small bowel showed acute ischaemic ulceration with focal infarction extending through the bowel wall and the appendix was normal.

## Discussion

Acute torsion of an appendage usually manifests as localised abdominal pain in one of the lower quadrants, since the sigmoid colon and caecum are the main sites of involvement. Mild fever and leucocytosis may develop, but symptoms of obstruction, nausea, vomiting and loss of appetite are infrequent. The clinical symptoms experienced by patients during the acute phase of torsion and infarction of appendices epiploicae will subside after several days or weeks. In such cases if the surgery is not performed, the infarcted appendage may undergo aseptic fat necrosis and gradually transform into a fibrotic or calcified mass. It may remain attach to the colonic serosa, or it may detach from it and remain as a mobile, loose body within the peritoneal cavity [4]. Ischaemia, which can be secondary to physiological or pathologic processes, is crucial in the pathogenesis of membranous fat necrosis, which is a distinct entity in appendices epiploicae [5]. Macroscopically, membranous fat necrosis in appendices epiploicae mimics nodal tuberculosis or metastatic tumour with necrosis and cystic change.

Primary epiploic appendagitis may occur as an acute primary process localised to one or several adjacent appendages or secondary to inflammatory changes originating in the colon or other neighbouring organs (eg. diverticulitis, Crohn’s colitis, acute pancreatitis, cholecystitis) [6]. Diverticulitis of the colon is by far the most common cause of secondary epiploic appendagitis, an association that arises from the close anatomic relationship between the colonic diverticula and appendices epiploicae.

Diagnostic laparoscopy for acute abdominal pain revealed gangrenous torsion in one of the appendices epiploicae in the ascending colon and sigmoid colon, which was excised with harmonic scalpel [7]. We tried laparoscopy in our case but it was impossible to continue as it had already progressed to the obstruction stage. It would have been useful to implement laparoscopy at an earlier stage. Radiology diagnosed a case in which torsion of an appendix epiploica led to the development of a pericolic abscess communicating between the colon and lesser sac [8].

All patients with acute diseases (inflammation, torsion, necrosis) of the appendices epiploicae should be treated by an emergency operation for removal of the appendices, closure of their beds with double-row interrupted sutures and reinforced with a pedicled omentum graft. Removal of the infarcted appendix results in cure, with no reported cases of recurrence. The disease can be fatal, four deaths having been reported in the literature [9]. Untreated, peritonitis or intestinal obstruction may result. Our case highlights the diagnostic delay and key to success is increased awareness of this entity in clinical practice.

## Conclusion

Acute torsion of an appendage usually manifests as localised abdominal pain in one of the lower quadrants. Untreated, peritonitis or intestinal obstruction may result. Heightened suspicion and use of diagnostic laparoscopy [10] would help to resolve the issue at an earlier stage and avoid major surgery as in our case. We suggest that evidence for epiploic appendagitis should be looked for if all the other organs are normal on diagnostic laparoscopy for unexplained abdominal pain.

## List of abbreviations

CT: Computed Tomography
US: Ultrasonography
